# Phylogenetic conservatism of environmental niches in mammals

**DOI:** 10.1098/rspb.2010.2207

**Published:** 2011-01-05

**Authors:** Natalie Cooper, Rob P. Freckleton, Walter Jetz

**Affiliations:** 1 Department of Ecology and Evolutionary Biology, Yale University, New Haven, CT 06520-8106, USA; 2 Department of Animal and Plant Sciences, University of Sheffield, Sheffield S10 2TN, UK

**Keywords:** environmental niche, temperature, precipitation, Brownian rate parameter, space, phylogeny

## Abstract

Phylogenetic niche conservatism is the pattern where close relatives occupy similar niches, whereas distant relatives are more dissimilar. We suggest that niche conservatism will vary across clades in relation to their characteristics. Specifically, we investigate how conservatism of environmental niches varies among mammals according to their latitude, range size, body size and specialization. We use the Brownian rate parameter, *σ*^2^, to measure the rate of evolution in key variables related to the ecological niche and define the more conserved group as the one with the slower rate of evolution. We find that tropical, small-ranged and specialized mammals have more conserved thermal niches than temperate, large-ranged or generalized mammals. Partitioning niche conservatism into its spatial and phylogenetic components, we find that spatial effects on niche variables are generally greater than phylogenetic effects. This suggests that recent evolution and dispersal have more influence on species' niches than more distant evolutionary events. These results have implications for our understanding of the role of niche conservatism in species richness patterns and for gauging the potential for species to adapt to global change.

## 1. Introduction

Phylogenetic niche conservatism (PNC) is the tendency of species to retain characteristics of their fundamental niche over time [1]. Recent work has highlighted the significance of PNC in understanding many biological patterns and processes [1–3]. For example, niche conservatism may explain species richness patterns at various scales and could reveal the role of ecology in speciation [1,2,4–6]. Most importantly, if high niche conservatism means that species will find it harder to evolve in the future, PNC may have consequences for conservation in the face of global change: all other things being equal, species with highly conserved niches may struggle to adapt to changing environments and could therefore face heightened risk of extinction under projected global change scenarios [3,7]. Species with more labile niches, on the other hand, may more readily cope with a locally changing climate and colonize or invade new areas [8], decreasing their risk of extinction.

PNC is predicted to occur because species inherit traits that determine their ecological niches (e.g. environmental tolerances) from their ancestors. Thus, closely related species are expected to have similar niches [9]. However, species that live in similar environments may also be ecologically similar because they experience similar environmental conditions [10]. This makes interpreting evidence of niche conservatism complicated. For example, two close relatives living in close proximity may be ecologically similar because they share a common ancestor, and hence the same inherited environmental tolerances. Alternatively, the species may live close to one another because they never dispersed far from their ancestral range. In this case, their trait similarity may reflect adaptation to the same environmental conditions, rather than inherited similarity [11]. This link between phylogeny and spatial distribution has implications for studies of niche conservatism because, if we ignore the spatial aspect, niche similarity may be wrongly attributed to common ancestry alone.

To date, evidence for PNC has been mixed and results seem to depend on the specific taxonomic group or niche variable studied (for recent reviews see [2,3,12]). For broad-scale environmental niches, the geographical scale (grain and extent) at which niches are analysed will be important. Moreover, results also depend on which method is used to measure PNC (e.g. [13]). Several methods exist, including comparisons of fossil and extant taxa (e.g. [14]), phylogenetic analyses to determine whether close relatives are more similar than expected under a Brownian motion model of evolution (e.g. [15]), the use of environmental niche models to investigate niche similarity (or equivalency) among related species (e.g. [5,16–18]), and methods for detecting phylogenetic inertia [19]. These methods produce different results, not only because of differences in methodology, but also because of the underlying assumptions each method makes about the definition of PNC and the mechanism by which it arises [20,21]. In order to interpret measures of PNC, both the method being used and the underlying mechanism quantified by the method must be clearly defined [20,21].

Most methods for detecting PNC are designed to test for conservatism in a single group of species; however, we are interested in comparing the degree of PNC in different groups. Recently, Ackerly [22] suggested that low rates of evolution in ecological niche variables could provide the best evidence of PNC in comparative data. Thus, a clade with a low rate of evolution for a given variable will contain species that have diverged less from one another, and therefore have more conserved trait values, than a clade with a higher rate of evolution. Here, we use the Brownian rate parameter, *σ*^2^, as a measure of the rate of evolution for various environmental niche variables. *σ*^2^ describes the rate at which the trait values of related species diverge from one another and it is equal to the rate of variance accumulation per unit of branch length [23,24]. A low value of *σ*^2^ for a clade implies that species' niche variables have not diverged much and thus the clade has a more conserved niche than another clade with a higher *σ*^2^ value.

Using this definition of PNC, we can form hypotheses about which traits may influence the degree of broad-scale environmental niche conservatism in a group. Firstly, climatic conditions are more homogeneous in the tropics [25]; therefore, we predict that tropical species will have more conserved environmental niches, and thus lower rates of niche evolution, than temperate species. This prediction has previously been used to explain why there are more species in the tropics, by suggesting that tropical species rarely disperse to temperate regions because they lack adaptations to survive cold temperate winters—the ‘tropical conservatism hypothesis’ [4].

Our second hypothesis is that species with small geographical ranges will show higher niche conservatism, and lower rates of niche evolution, than species with large ranges. This is because narrowly distributed species will, on average, occupy a narrower range of climatic conditions, experience less temporal and spatial environmental variability and exhibit fewer local adaptations among populations across their range, potentially facilitating evolutionary conservatism of broad-scale environmental associations [26,27]. Populations of narrow-ranged species also tend to face relatively smaller geographical variation in predators, prey or other biotic factors [28], potentially resulting in tighter environmental associations [29]. For our third hypothesis, we predict that dietary and habitat specialists will have more conserved environmental niches, and lower rates of niche evolution, than more generalist species. Both types of specialization are inherently linked to environmental specialization of some sort, which in turn suggests conserved climatic associations. Obviously, these factors are interconnected: tropical species tend, on average, to have smaller geographical ranges than temperate species and harbour more specialists who are also likely to have narrower geographical ranges [26]. These variables are also positively correlated with body size (e.g. [30]); therefore we also hypothesize that small species will have more conserved niches than large species.

As outlined above, PNC is expected to have both phylogenetic and spatial components: close relatives are usually similar because they share a recent common ancestor [9], but species living in close geographical proximity are also expected to be similar because they experience similar environmental conditions [10]. Spatial autocorrelation is particularly important in our analyses because dispersal limitations alone cause closely related species to occupy nearby regions, and environmental variables tend to have a very strong spatial structure [31,32]. An apparent phylogenetic signal in environmental niches may thus arise owing to spatial proximity alone, in the absence of a strong effect of shared phylogenetic history [11]. In order to understand the relative importance of species' distributions and phylogenetic relationships to niche conservatism, we use a method that can account for the spatial and phylogenetic components of trait evolution simultaneously [11].

We use mammals as our study group because there are ecological and life-history data for most extant species (e.g. [33]), and a comprehensive estimate of mammalian phylogeny is available [34,35]. We expect lower rates of niche evolution, and thus lower *σ*^2^ values, in the subgroups that are predicted to show greater levels of niche conservatism. Specifically, we predict lower *σ*^2^ values in tropical, small-ranged, small and specialized mammals compared with temperate, large-ranged, large and generalist species. We test these hypotheses by estimating *σ*^2^ for various broad-scale environmental niche variables, and determine the relative effects of space and phylogeny on PNC. As far as we are aware, this is the first attempt to quantify how different species' attributes may relate to the degree of niche conservatism in a group of this size.

## 2. Material and methods

### (a) Data

We used species-level geographical range maps from the IUCN global mammal assessment [36] linked to global climate layers to derive species' broad-scale environmental niches (Grinnellian niche [37]). We overlaid these range maps with a 110 × 110 km equal area grid in Behrman projection and used grid cell occurrence to extract environmental conditions from a variety of global layers. We extracted the following environmental variables as the mean value (‘environmental centroid’ [38]) across each species's range: log mean annual precipitation (mm), log mean precipitation of driest month (minimum precipitation; mm), within-year variation in precipitation (standard deviation of log monthly precipitation values), log mean annual temperature (°C), log mean temperature of coldest month (minimum temperature; °C) and within-year variation in temperature (standard deviation of log monthly temperature values). Temperature and precipitation estimates were based on the University of East Anglia's Climatic Research Unit gridded monthly climatology 1961–1990 dataset at native 10 min resolution [39]. We transformed all variables so they had a mean of 0 and variance of 1 to allow the Brownian rate parameter, *σ*^2^, to be compared among groups (see below).

We defined species as tropical if their geographical range centroid was within the tropics and temperate if their geographical range centroid was outside the tropics. Some species occur in both tropical and temperate regions, so, to determine whether this influenced our results, we also identified species that only occurred in the tropics (i.e. maximum latitude less than 23.4° and minimum latitude greater than −23.4°) and species that only occurred in temperate regions (i.e. maximum latitude less than −23.4° and minimum latitude greater than 23.4°). We defined species having large and small geographical range sizes as those in the fourth and first quartile, respectively, of the overall mammalian geographical range size distribution (large range greater than 1388 × 10^3^ km^2^; small range less than 34.5 × 10^3^ km^2^).

Using body mass data from the PanTHERIA database [33], we defined large and small species as those in the fourth and first quartile, respectively, of the overall mammalian body size distribution (large > 992.4 g; small < 24.93 g). We defined specialization as the number of dietary items eaten multiplied by the number of habitats occupied also using data from PanTHERIA [33]. Specialized species were those in the first quartile of our specialization variable and generalist species were those in the fourth quartile (specialists ≤ 3; generalists ≥ 6). We used the ‘best dates’ supertree of Bininda-Emonds *et al*. [34,35] as our phylogeny (see below). Freckleton & Jetz's [11] method requires information on the spatial distance between each pair of species so we used the geodesic distance between species' geographical range centroids.

### (b) Analyses

We first estimated *φ*, *λ*′ and *γ* ([11]; R code available from R.P.F. on request) for all species in the phylogeny using each environmental variable in turn. In these models *φ* measures the relative contribution of phylogenetic and spatial effects, and varies between zero (where there are only phylogenetic effects) and one (where there are only spatial effects). *λ*′ is a spatially corrected version of Pagel's *λ* [40], the multiplier of the off-diagonal elements of a phylogenetic variance covariance matrix, which best fits the data [41]. *λ*′ is equal to (1−*φ*)*λ* and varies from zero (where trait values are independent of phylogeny) to one (where trait values are structured according to a Brownian motion model of trait evolution). Finally, *γ* represents the proportion of trait variation (which is independent of both space and phylogeny) and is calculated as (1−*φ*)(1−*λ*). In terms of PNC, *φ* will be high when trait similarity among close relatives is due to their similar geographical distributions, rather than their phylogenetic relatedness, and spatial effects have a large influence on trait evolution. Conversely *λ*′ will be high when close relatives are similar owing to their evolutionary history, rather than their spatial proximity.

Practically, we used maximum likelihood to search for the optimum values of *φ* and *λ* simultaneously, by maximizing the following likelihood equation (note that both *φ* and *λ* are constrained to be between 0 and 1).2.1
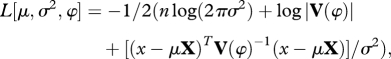
where **V** is equal to2.2
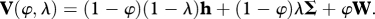


In these equations (eqns (2.4) and (2.6) in [11]), *μ* is the weighted mean of the trait at the basal node, *σ*^2^ is the variance parameter (both *μ* and *σ*^2^ are estimated assuming a multivariate normal distribution of trait values at the tips of the tree), *x* is the data, **X** is the design matrix, **V** is the expected variance–covariance matrix for the variable in question, ***Σ*** is the variance–covariance matrix of the phylogeny, **h** is a vector containing the heights of the tips of the tree (i.e. the leading diagonal of ***Σ***) and **W** is the variance–covariance matrix of spatial distances among species' geographical range centroids. Both **V** and **W** are calculated using independent contrasts (see [23] for details of the algorithm used). In terms of **W**, this assumes that spatial distance accumulates with distance away from the root of the phylogeny. For more details of this method see [11].

There are several methods that test for variation in rates among groups of species (e.g. [24,42]); however, these methods require that each node in the phylogeny is assigned to one of the groups being compared. For example, if rates in temperate and tropical species were compared, ancestral state reconstruction would be used to define each branch in the phylogeny as either temperate or tropical. However, there is a debate about the usefulness of these ancestral state reconstructions. They are often ambiguous (and sometimes misleading) and without additional fossil evidence they are problematic for ascertaining the geographical locations of ancestral species. Therefore, we instead used the Brownian rate parameter, *σ*^2^, as our measure of rate and determined the significance of any differences among groups using simulations (see below). Using our method, some internal branches will lead to species from both of the groups being compared and these branches will therefore be used to estimate *σ*^2^ in both groups (e.g. internal branches that lead to a family containing both temperate and tropical species will be included in the *σ*^2^ estimates for both temperate and tropical species). Consequently, the variances of the groups cannot be compared using parametric methods that assume independence, such as an *F*-ratio test. To compare variances we therefore used a simulation approach to test for differences among groups.

In order to compare the degree of niche conservatism among groups, we first pruned the phylogeny so it only contained the species within the group in question (e.g. only tropical species). We then used the *λ*′ value (estimated above) for the first environmental variable (for the group in question) to transform the phylogeny, before estimating the Brownian rate parameter, *σ*^2^, for that environmental variable. Note that *λ*′ (and *λ*) transformations scale the internal branch lengths of the phylogeny relative to the external branch lengths and then add 1−*λ*′ (or *λ*) times the total tree height to the external branches. *σ*^2^ was estimated as the sum of the standardized independent contrasts for the pruned phylogeny squared, then divided by the number of species in the pruned phylogeny (note that this yields the same value as for the unbiased estimator of [24]). This procedure was repeated for each of the environmental variables in turn and then for each of the other groups (i.e. tropical, temperate, large range, small range, large, small, generalist or specialist species), as well as for all the species in the phylogeny. We also performed these analyses by transforming the phylogeny using an estimate of *λ*, rather than *λ*′, to determine whether removing the spatial aspects of phylogenetic signal affected our results. Note that *σ*^2^ cannot be compared across trees that have been differently scaled; however, because all trees for a given trait were scaled in the same way, we can compare rates across trees for each trait (although we cannot compare rates among different traits).

We used simulations to determine whether differences in *σ*^2^ values among groups were significantly greater than expected by chance. First, for a chosen environmental variable (e.g. mean precipitation), we used the value of *λ*′ (or *λ*) estimated above for the environmental variable (across all the species in the phylogeny) to transform the whole phylogeny. We did this in order to ensure that the amount of phylogenetic signal in the simulated data was the same as the amount of signal in the actual environmental data. We then simulated data along the *λ*′ (or *λ*) transformed phylogeny using a constant rate Brownian motion model (similar to simulations in [43]). Next, for each comparison in turn (e.g. tropical versus temperate species), we pruned the phylogeny to the correct subsets of species and estimated *σ*^2^ values for each group. We calculated the ratio of the *σ*^2^ values as the larger *σ*^2^ value divided by the smaller *σ*^2^ value.

We repeated this procedure 1000 times to get a distribution of simulated *σ*^2^ ratios for the comparison and environmental variable in question. We then applied the procedure to each comparison and each environmental variable in turn to obtain a simulated distribution for each combination. We then compared the appropriate simulated distributions of *σ*^2^ ratios to the observed *σ*^2^ ratio (e.g. the observed *σ*^2^ ratio for mean precipitation in the tropical versus temperate comparison was compared with the simulated distribution for mean precipitation in the tropical versus temperate comparison). If the observed *σ*^2^ ratio was greater than in 99.9 per cent of the simulated *σ*^2^ ratios, the difference was considered significant (*α* = 0.001).This simulation approach accounts for non-independence of estimates of *σ*^2^ in the compared groups, and ensures that type I errors will be minimized. The approach is not as powerful as that described by Thomas *et al*. [42] as it does not include information on the ancestral states of the differentiating variable; however, as argued above, it may not be meaningful to attempt such ancestral state reconstructions for the variables we are studying.

One factor that could influence our results is that the phylogeny is not fully resolved. If polytomies tend to result in a decrease in the mean height of the root of internal nodes, then the rate of evolution will be underestimated [44]. Thus, if polytomies are not spread evenly across the two groups being compared, any differences in rate may be the result of differences in phylogenetic resolution rather than PNC. Unfortunately resolution varies among groups (tropical = 47.51%; temperate = 57.94%; large range = 67.01%; small range = 51.65%; large = 76.98%; small = 49.29%; generalist = 75.45%; specialist = 66.08% resolved), so in order to determine whether this was an issue we repeated all the analyses above using a fully resolved phylogeny. The polytomies in this phylogeny were resolved randomly by removing all but two of the species (or nodes for internal polytomies) in each polytomy. We used R v. 2.10.1 in all analyses [45].

## 3. Results

**Figure 1.**
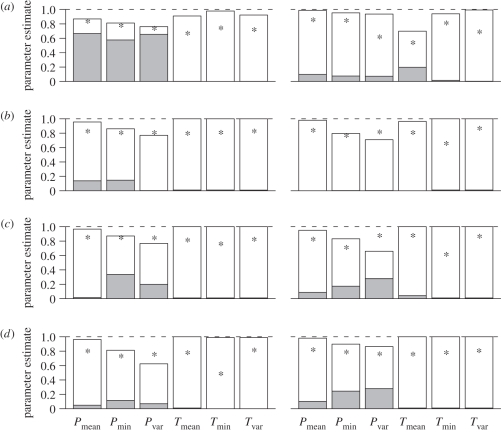
Bar charts showing estimates of *φ* (white) and *λ*′ (grey) for each environmental variable in (*a*) temperate and tropical, (*b*) large- and small-geographical ranged, (*c*) large- and small-bodied, and (*d*) generalist and specialist mammals. *P*, precipitation; *T*, temperature; min, minimum; var, variability. Asterisk denotes value of non-spatially corrected *λ*. The dotted line indicates the maximum value of each parameter. Note that since *φ*, *λ*′ and *γ* sum to one, there is no need to display the *γ* values.

Estimated *φ*, *λ*′ and *γ* values for all species in the phylogeny were as follows: mean precipitation: *φ* = 0.852, *λ*′ = 0.129, *γ* = 0.019; minimum precipitation: *φ* = 0.717, *λ*′ = 0.196, *γ* = 0.087; precipitation variability: *φ* = 0.662, *λ*′ = 0.168, *γ* = 0.170; all temperature variables: *φ* = 0.990, *λ*′ = 0.010, *γ* < 0.001 (using a fully resolved phylogeny: mean precipitation: *φ* = 0.764, *λ*′ = 0.213, *γ* = 0.023; minimum precipitation: *φ* = 0.661, *λ*′ = 0.297, *γ* = 0.092; precipitation variability: *φ* = 0.557, *λ*′ = 0.210, *γ* = 0.234; all temperature variables: *φ* = 0.990, *λ*′ = 0.010, *γ* < 0.001). These *φ* values are much higher than *λ*′ values, indicating that spatial effects on the environmental variables were greater than the purely phylogenetic effects. *φ* and *λ*′ values for the three groupings in this study are shown in figure 1 (note that since *φ*, *λ*′ and *γ* sum to one, there is no need to display the *γ* values, so we omit them to simplify the figures; electronic supplementary material, appendix A, figure A1 shows the results using a fully resolved phylogeny that excludes species with polytomies). Across the four subgroups and all six variables, values of *φ* are generally much higher than values of *λ*′, except for precipitation variables in temperate species where *λ*′ values are higher (figure 1; electronic supplementary material, appendix A and figures A1 and A2). We note that simultaneously accounting for spatial non-independence yields dramatically lowered estimates of the phylogenetic signal than if *λ* was quantified non-spatially (figure 1). *λ* and *λ*′ are correlated but not perfectly (all variables, 16 orders: *ρ* = 0.252; *p* = 0.013).

Across the three groupings, several clear differences in *σ*^2^ emerged (table 1). Tropical species had lower *σ*^2^ values than temperate species for all variables except minimum precipitation, although this difference was not significant for precipitation variability. Small-ranged species had significantly lower *σ*^2^ values than large-ranged species for all temperature variables but higher values for precipitation variables. Small-bodied species had lower *σ*^2^ values than large-bodied species for all variables except temperature and precipitation variability. However, only the difference in mean temperature was significant. Specialist species had lower *σ*^2^ values than generalist species for all variables, but the differences in minimum precipitation and precipitation variability were not significant. These differences among variables suggest that PNC studies that use complex abstractions of a number of variables (e.g. [16]) may fail to find evidence of niche conservatism because the signal from one variable may be masked by the opposite responses of other variables. Results using *λ* rather than *λ*′ to transform the phylogeny before estimating *σ*^2^ are qualitatively similar (electronic supplementary material, appendix A and table A1); where there are differences they are usually non-significant, except for precipitation variability in both the tropical/temperate and specialist/generalist comparisons. Results for an alternative definition of tropical and temperate species are also qualitatively similar (except for precipitation variability in the *λ* analyses; electronic supplementary material, appendix A, tables A2 and A3 and figure A2), as are results for analyses using a fully resolved phylogeny (except for minimum temperature in the body size comparison; electronic supplementary material, appendix A, table A4 and figure A1).

**Table 1.** Brownian rate parameter (*σ*^2^) values estimated for each environmental variable for all mammalian species in the phylogeny and four groups, after transforming the branch lengths in the phylogeny by the appropriate value of *λ*′. *P*, precipitation; *T*, temperature; min, minimum; var, variability. *n* = number of species (note that this varies between the mean and minimum variables and the variability variables). *σ*^2^ values are equal to *σ*^2^ × 1000. *σ*^2^ values in bold are significantly lower than *σ*^2^ values for the other group (*α* = 0.001).

group		*n*	*P*_mean_		*P*_min_		*T*_mean_		*T*_min_		*n*	*P*_var_		*T*_var_	
			*λ*′	*σ*^2^	*λ*′	*σ*^2^	*λ*′	*σ*^2^	*λ*′	*σ*^2^		*λ*′	*σ*^2^	*λ*′	*σ*^2^
all species	—	3511	0.129	5.102	0.196	5.634	0.010	5.928	0.010	6.103	3720	0.168	6.012	0.010	6.019
latitude	temperate	1204	0.665	7.788	0.576	**5.346**	0.001	7.477	<0.001	8.609	1240	0.654	7.883	0.001	5.594
	tropical	2371	0.098	**3.861**	0.075	7.062	0.197	**0.990**	0.015	**0.469**	2548	0.072	7.233	0.003	**0.845**
range size	large	968	0.138	**4.693**	0.147	**3.709**	0.010	9.131	0.010	10.41	970	0.002	**5.723**	0.010	8.675
	small	640	<0.001	6.241	0.002	7.553	<0.001	**3.947**	0.010	**3.111**	842	0.003	6.729	0.010	**3.458**
body size	large	691	0.014	5.325	0.337	6.050	0.010	6.863	0.010	6.671	708	0.198	6.052	0.010	5.495
	small	688	0.087	5.274	0.173	5.293	0.043	**5.003**	0.010	5.751	702	0.278	6.910	0.010	6.263
specialization	generalist	402	0.049	6.781	0.115	5.876	0.010	6.745	<0.001	6.571	411	0.070	5.609	<0.001	7.504
	specialist	874	0.102	**4.617**	0.244	5.027	0.010	**5.433**	0.010	**5.202**	906	0.280	5.113	0.010	**5.038**

## 4. Discussion

The degree of niche conservatism in mammals varied among groups of species: tropical, small-ranged and specialist species had more conserved temperature niches than temperate, large-ranged or generalist species. These results fit our predictions: tropical species are expected to show high levels of temperature niche conservatism (e.g. [4]), and both small-ranged and environmentally specialized species experience less temporal and spatial environmental variability, which should lead to evolutionary conservatism of their broad-scale environmental niches [26,27]. These differences were not merely the result of body size differences because small species only had significantly more conserved temperature niches than large species for one temperature variable.

Interestingly, when we partitioned niche conservatism into spatial (*φ*) and phylogenetic (*λ*′) components, we found that values of species' environmental niche variables were predominantly driven by spatial effects (*φ* > *λ*′). This does not mean that phylogeny plays no part in determining species' niches; indeed, spatial and phylogenetic effects are expected to be closely linked. Close relatives will tend to live in similar places unless they have dispersed rapidly away from their ancestral ranges and traits can have high phylogenetic signals even if there is a large degree of spatial autocorrelation [11]. Furthermore, the method used here assumes that spatial distances between species pairs evolve along the phylogeny [11]. Instead, this result probably reflects the relatively greater influence of recent evolutionary events and current species distributions on species' environmental niches, compared with the influence of evolutionary events deeper in the phylogeny. These results may have implications for studies that estimate phylogenetic signal in environmentally correlated variables.

High levels of thermal niche conservatism are thought to increase the risk of extinction for species under global change scenarios [7]. All else being equal, our results suggest that under future global warming tropical, small-ranged and specialist species may be particularly strongly at risk. Actual future risk will be modified by a multitude of additional broad- and fine-scale factors, including the geography of projected warming (larger away from the equator) and anthropogenic land-use change (more intense at low latitudes) [46]. Unfortunately, other extinction drivers—such as overexploitation and habitat loss—also disproportionately influence tropical, small-ranged and specialized mammals [47]; thus, our findings about thermal niche conservatism suggest that climate change may make an already bad situation worse.

This interpretation, however, makes a number of assumptions. Firstly, we assume that the pattern of PNC is the result of the species's environmental tolerances, yet the pattern could equally be due to dispersal limitations (which could also explain why spatial effects over-ride phylogenetic effects) or some other factor. Secondly, we also assume that niche variables reflect the conditions in which the species can survive, whereas in reality they reflect where the species currently lives (i.e. its realized niche) [37]. It is probable that species can survive in a much broader range of conditions but are restricted by other abiotic or biotic factors [37]. Thirdly, we assume that all areas will be equally influenced by climate change, but current projections suggest that temperate areas will experience much greater temperature changes than the tropics [48]. Therefore, niche conservatism in tropical species may only be problematic for species with very restricted thermal tolerances, especially given the homogeneous nature of temperature in tropics, which should provide areas of stable temperature [25]. Note that this may also partially account for the differences among temperate and tropical species: two randomly selected close relatives in the tropics will have more similar thermal niches than in the temperate zone simply because temperature is more uniform across the tropics. Finally, we assume that thermal niche conservatism means that species will not respond to changes in climate. However, as described above, the high values of *φ* we found suggest that recent events have played a greater role in shaping species' niches than historical events. This indicates that species' niches have responded to (relatively) recent changes in their environment, perhaps by shifting their geographical ranges to track their niches through time. These kinds of range movements in response to temperature changes have already been observed in several mammalian species (e.g. [49–51]). If mammals are able to shift their geographical ranges then temperature changes will only begin to drastically increase the levels of mammalian extinction risk when barriers (e.g. mountains or the sea), or other abiotic or biotic (e.g. competition and predation) limitations, prevent species from tracking suitable habitat. Thus, species found in areas with many range-limiting features (i.e. areas of high landscape impermeability [52]) may be especially at risk.

Niche conservatism may also be important in driving the contemporary latitudinal species richness gradient [4,53–55]. According to the ‘tropical conservatism hypothesis’, most species arise in the tropics, but their inability to adapt to cold winters prevents them from dispersing into the temperate zone. Thus, there are more species in the tropics compared with temperate regions because of temperature niche conservatism [4]. Our results support this hypothesis in mammals: tropical mammals had more conserved temperature niches than temperate species. We also found that temperate species had more conserved minimum precipitation niches than tropical species, which suggests that conservatism in precipitation niche could prevent any mammalian group that originated outside the tropics from dispersing there from temperate regions. Thus, niche conservatism may also account for species richness patterns in groups that do not have an extra-tropical diversity peak—an explanation also given for the inverse latitudinal richness gradient seen in some New World snakes [56].

Obviously our methods have limitations and make a number of assumptions. The definition of a species's environmental niche is naturally fraught with difficulty. Here, we use the species's geographical range to derive an estimate for the environmental niche. However, this makes the assumption that the distribution of a species is a true representation of its fundamental niche. In reality, it (imperfectly) reflects the species' realized niche and is influenced by not only environmental tolerances but also by dispersal limitations and biotic variables such as predation and competition [37] (the relative importance of which may vary according to whether species are tropical or temperate). This assumption is common to all analyses of this kind (e.g. [16]). In addition, given the limited spatial accuracy of expert range maps [57], species-typical environments needed to be quantified at relatively coarse grain, while of course environmental predictors of species' distribution are not scale-invariant [58]. Additionally, the degree to which geographical ranges (and with them simple measures of their realized niche) reflect an approximation of species' actual environmental tolerances or fundamental niches, may be geographically non-random. However, we expect the coarse grain and global extent of our analyses to help address these issues, as broad signatures rather than correlates at fine scale (where biotic effects often dominate) are quantified. Furthermore, mean values of environmental variables clearly represent a simpler quantification of species' environmental niches than, for example, parameters derived from niche modelling. Niche modelling results may be strongly dependent on methodology and user decisions so, for the purpose of this first analysis, our use of centroid values seems transparent, powerful and sound [38]. However, neither these centroid values nor parameters derived from niche models are true physiological or life-history variables, so these analyses may be oversimplified. Ideally, we would use the critical maximum or minimum values of the variables for each species, but these data are not available. Improvements may also be possible on measurement of the geographical distance between two species, which here we simply defined as the distance between their geographical range centroids. Finally, the way we divided species into binary groupings was fairly crude, particularly our definition of specialized species. We used the best data available for a large number of mammals (i.e. PanTHERIA's diet and habitat data [33]), however, specialization is likely to be at a much finer scale than these data and may differ depending on the trait examined.

Our results show that the degree of niche conservatism in mammals varies among tropical and temperate, large-ranged and small-ranged, and generalist and specialist species. Spatial effects on niche variables were generally larger than purely phylogenetic effects, suggesting that recent evolution and current species distributions have a greater influence on species' niches than more distant evolutionary events. These differences in the degree of niche conservatism among groups may have implications for our understanding of species richness patterns and conservation in a changing world.
